# Is there “seasonal” variation in height velocity in children treated with growth hormone? Data from the National Cooperative Growth Study

**DOI:** 10.1186/1687-9856-2013-2

**Published:** 2013-02-02

**Authors:** Dorothy I Shulman, James Frane, Barbara Lippe

**Affiliations:** 1 Department of Pediatrics, University of South Florida Morsani College of Medicine, MDC 62, , 12901 Bruce B. Downs Blvd.,Tampa, FL, 33612, USA; 2 Biostatistical Consultant, Santa Monica, CA, USA; 3 Consultant Genentech, Inc., South San Francisco, CA, USA

**Keywords:** Seasonal growth, Growth hormone deficiency, Children

## Abstract

**Background:**

Growth rate In children is reported to have seasonal variability. There are fewer published data regarding seasonal variability while on growth hormone (GH) therapy, and none analyzing growth rate with respect to number of daylight hours.

**Methods:**

We analyzed 11,587 3-month intervals in 2277 prepubertal children (boys ages 3–14 years, girls ages 3–12 years) with idiopathic GH deficiency from the National Cooperative Growth Study (NCGS) database. All were naive to recombinant human GH (rhGH) therapy. Data were submitted from 31 US study centers. Seasonal variation in height velocity (HV) was assumed to be associated with the average number of daylight hours during the interval of time over which HV was computed. Number of daylight hours was determined from the date of the measurement and the latitude of the study center. Other independent variables evaluated included: height standard deviation score (SDS) at the beginning of the interval, chronologic age at the beginning of the interval, time from the start of rhGH treatment to the middle of the interval, month of the year, body mass index SDS at the beginning of the interval, rhGH dose/kg, mother’s height SDS, father’s height SDS, and log base 10 of the maximum stimulated GH concentration.

**Results:**

All variables examined, except month of the year, correlated significantly with interval HV**.** There was significant “seasonal” variability at all latitudes, with summer annualized HV being greater than winter HV. This difference was greatest in the first year of therapy (0.146 cm/yr/daylight hour; *P* < 0.0001) but persisted in subsequent years (0.121 cm/yr/daylight hr; *P* < 0.0001). The difference increased with distance from the equator. Growth rate over the year was not different among the latitudes reflected in this North American study.

**Conclusions:**

There is “seasonal” variation in growth of children on rhGH therapy that correlates with number of daylight hours. The effect is modest and is greatest in the first year of therapy. Annual growth rate appears to be equal in children among latitudes covered by the US consistent with exposure to an equal number of daylight hours over the year. The physiologic mechanism behind this seasonal variation is not yet understood.

## Background

Growth rate in children varies during the calendar year, with a faster rate of growth during the spring/summer than in the winter [1,2]. “Seasons” per se are not fixed throughout the world, as definitions vary as to whether they are meteorologically defined, culturally defined by holidays, or socially defined by the school year. Nevertheless, the most constant definitions focus on warmth and sunlight with the most reproducable mathematical variable for seasonality being the number of daylight hours. “Seasonal” variation has been evaluated to a lesser extent in children on growth hormone (GH) therapy [3-5] and has not specifically been evaluated with respect to number of daylight hours. As the National Cooperative Growth Study (NCGS) database now contains 220,000 patient-years of growth data on children receiving recombinant human growth hormone (rhGH) therapy, we asked the following questions: 1) Does exogenous rhGH obscure “seasonal” variability in height velocity (HV) in prepubertal children with isolated growth hormone deficiency (IGHD) in North America? 2) Is the magnitude of the “seasonal” difference in HV enough to influence clinical decision making regarding assessment of efficacy of a short trial of rhGH therapy? 3) Are there differences in annual HV in treated children with IGHD living at northern and southern latitudes in the United States?

## Methods

The NCGS database was initiated in December 1985 to collect data in children treated with rhGH for evaluation of safety and efficacy. Anonymous data were entered by clinical investigators in the US including date of birth, sex, height, weight, etiology of short stature, peak serum GH response to stimulation testing, Tanner pubertal stages, parental heights, and GH dose for patients treated with Genentech’s rhGH products. The database reflects 220,000 rhGH treatment years.

### Patients

Analysis was performed using 2277 patients with IGHD (peak GH response to stimulation testing <10 ng/mL) chosen from 31 US clinical study centers in the NCGS database spanning the range of latitudes in the United States. Patients were GH naive, boys were restricted to ages 3 to 14 years and girls to ages 3 to 12 years, and prepubertal during the time intervals for which HV was assessed. Duration of treatment range was 3 months to 10 years.

### Analysis of seasonal variation in HV

Seasonal variation in HV was assumed to be associated with the average number of daylight hours during the interval of time over which HV was computed. The number of daylight hours depends both on date and latitude. Latitude was assumed to be that of the study site. HV was assessed for 11,587 3-month intervals for these 2277 patients. The average number of daylight hours was computed for each interval. Other independent variables included: height standard deviation score (SDS) at the beginning of the interval, chronologic age at the beginning of the interval, time from the start of rhGH treatment to the middle of the interval, month of the year, body mass index (BMI) SDS at the beginning of the interval, rhGH dose/kg at the beginning of the interval, mother’s height SDS, father’s height SDS, and log base 10 of the maximum stimulated GH concentration.

In NCGS, there was no required frequency for the measurement of heights. For each patient, 3-month HV was computed for each time interval between height measurements for which the interval length was between 2 and 4 months, i.e., between approximately 60 days and 120 days. Thus, if the time interval between two successive heights for a patient was greater than 120 days, then no HV for that time interval was used. For example, if the time from baseline for measurement of heights for a patient was 30, 89, and 180 days, then HVs were computed for the interval from baseline to day 89 and for the interval from day 89 to day 180. In order to distinguish the seasonal effect from the effect of daylight hours, the month of the year was added to the regression model for the 3-month HVs during the first year of therapy. Specifically, the month of the year for the mid-point of the time interval for each HV was used. (For example, if a “3-month” time interval started on April 3 and ended on July 10, then the mid-point would be May 22, so the month for the mid-point would be May.)

Because multiple measurements were used for each patient, the SAS mixed models procedure was used to perform the analysis. The analysis was performed using the June 2011 version of the NCGS database.

## Results

Results are summarized in Tables 1 and 2. Each of the independent variables evaluated, except month of the year, contributed significantly to the prediction of the 3-month interval HV during the first year. The regression coefficient for the average number of daylight hours was 0.146 cm/yr per daylight hour in year 1 of rhGH treatment, and 0.121 cm/yr per daylight hour in subsequent treatment years (*P* < 0.0001). The overall average was 0.133 cm/yr per hour of daylight (*P* < 0.0001). In the multiple regression analysis there was no statistical relationship between month of the year and the 3-month interval growth rate (*P* = 0.65), while the average number of daylight hours during the interval remained highly statistically significant.

**Table 1:** 3-Month height velocity during the first year of treatment and regression coefficients (2090 Patients, 5033 HVs)

	**Mean (SD)**	**Regression coefficent**	***P*value**
3-month HV during 1st year (cm/yr)	9.7 (3.7)		
Daylight hours	11.9 (1.7)	0.146	<0.0001
Age (yr) at start of 3-month interval	9.0 (2.8)	−0.22	<0.0001
Height SDS at start of 3-month interval	−2.2 (0.8)	0.28	<0.0001
BMI SDS at start of 3-month interval	−0.4 (1.1)	0.52	<0.0001
Mother’s height SDS	−0.6 (1.1)	0.19	<0.0001
Father’s height SDS	−0.4 (1.1)	0.10	0.0340
Maximum stimulated GH (ng/mL)	6.1 (2.4)	−2.1*	<0.0001
GH Dose (mg/kg/wk) at start of 3-month interval	0.30 (0.05)	7.9	<0.0001
Time from GH start to midinterval for HV (yr)	0.4 (0.3)	−3.57	<0.0001

*Using log base 10 of maximum stimulated GH response.

GH, growth hormone; HV, height velocity; SD, standard deviation; SDS, standard deviation score.

**Table 2:** 3-Month height velocity after the first year of treatment and regression coefficients (1292 Patients, 6356 HVs)

	**Mean (SD)**	**Regression coefficient**	***P*value**
3-month HV after 1st year (cm/yr)	7.2 (2.9)		
Daylight hours	11.9 (1.7)	0.121	<0.0001
Age (yr) at start of 3-month interval	9.2 (2.1)	−0.076	<0.0001
Height SDS at start of 3-month interval	−1.4 (1.0)	0.16	0.0002
BMI SDS at start of 3-month interval	−0.1 (1.0)	0.34	<0.0001
Mother’s height SDS	−0.6 (1.2)	0.03	0.3434
Father’s height SDS	−0.4 (1.2)	0.02	0.6048
Maximum stimulated GH (ng/mL)	6.1 (2.4)	−1.4*	<0.0001
GH dose (mg/kg/wk) at start of 3-month interval	0.32 (0.07)	1.6	0.0053
Time from GH start to midinterval for HV (yr)	2.8 (1.5)	−0.45	<0.0001

*Using log base 10 of maximum stimulated GH response.

GH, growth hormone; HV, height velocity; SD, standard deviation; SDS, standard deviation score.

**Figure 1:**
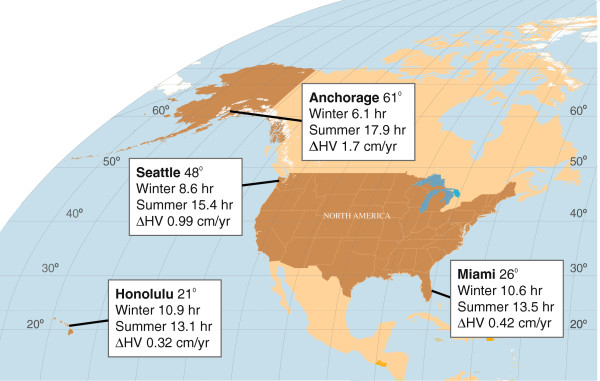
Effect of daylight hours on height velocity (HV).

Examples of the effect on number of daylight hours on annualized HV from some of the clinical sites are provided in Figure 1.

In Seattle, Washington, at 48° latitude, the average number of daylight hours during the 3 months of the year with the shortest days (roughly November 6 to February 5) is 8.6 hours and during the 3 months with the longest days (roughly May 6 to August 6) is 15.4 hours; the mean difference in annualized HV between these 2 periods during the first year of treatment is 0.99 cm/yr (6.8 hr × 0.146 cm/yr/daylight hour).

In Miami, Florida, at 26° latitude, the average number of daylight hours during the 3 months of the year with the shortest days is 10.6 hours, compared with 13.5 hours during the longest days; the mean difference in the annualized HV in the first year is 0.42 cm/yr in the first year.

In Anchorage, Alaska, at 61° latitude, average number of daylight hours during the 3 months of the year with the shortest days is 6.1 hours, compared with 17.9 hours during the longest days; the difference in the annualized HV between winter and summer is 1.7 cm/yr in the first year.

In Honolulu, Hawaii, at 21° latitude, the site closest to the equator, the difference between the daylight hours between the 3 months of winter with the shortest days (10.9) and those of summer with the longest days (13.1) was the smallest among the illustrated sites, and the difference in annualized HV between winter and summer the smallest as well, 0.32 cm/year.

There was no effect of latitude on HV measured over the entire first year (*P* = 0.40). BMI SDS was unrelated to the number of daylight hours (*P* = 0.28).

The role of peak stimulated GH concentration on the magnitude of the effect of daylight hours on 3-month HVs during the first year of GH therapy was analyzed. Of 2277 patients, only 342 had maximum stimulated GH ≤ 3 ng/mL (includes 5033 3-month HV intervals). In this analysis, the effect of peak GH was slightly statistically significant (*P* = 0.027). The regression coefficient for daylight hours for patients with peak stimulated GH ≤ 3 ng/mL was 0.183 and was 0.140 for those with a response > 3 ng/mL but less than 10.

## Discussion

Seasonal variation in growth has been reported for normally growing children by several authors, most reporting peak growth during the summer months in the Northern Hemisphere [1-3]. Ikeda and Watanabe did not identify seasonal variation in growth of 1000 Japanese school children ages 6–11 years studied between 1983 and 1984 [6]. They attributed this to the use of modern home-heating systems. Lee studied 27 boys and girls in Oakland, California, and observed persistent seasonal variability despite living in a climate that had little variation in temperature and humidity throughout the year, suggesting that temperature was not an important factor [2].

Number of daylight hours is thought to play a role in variation of seasonal growth rates, although the mechanism is not understood. Blind children have been reported to have limited variation in growth rate throughout the year [7]. Rudolf et al. performed GH stimulation testing and integrated 24-hour GH profiles in 84 normally growing children in 2 consecutive seasons and identified no differences in GH or insulin-like growth factor 1 (IGF-1) levels to account for the observed variation in growth rate [5]. These authors also evaluated growth in 52 GH-deficient children and identified seasonal variabitity in growth even on GH therapy. Growth rate generally peaked in summer, and was lowest in the Israeli autumn. They concluded that the effect of daylight hours appears to not be mediated through GH secretion.

In an early NCGS summary report, Tiwary noted seasonal variation in GH-treated patients with idiopathic short stature, IGHD, and Turner syndrome [3]. In our study, 3-month HV data have been analyzed in a large number children diagnosed with IGHD children who were initially naive to GH therapy. Seasonal variability in HV during rhGH therapy was associated with number of daylight hours and was most apparent at latitudes farthest from the equator. The effect of daylight hours appears greatest in the first year but persists throughout subsequent years of therapy. The effect is modest and is likely to affect clinical assessment of short-term growth rate (3 to 4 months) only in latitudes far from the equator, particularly if there are additional contributing factors, such as intercurrent illness or intermittent compliance. Effect of daylight hours is slightly greater in children with peak stimulated GH response ≤ 3 ng/mL, however, the significance of this obervation is unclear due to small sample size.

Interestingly, annual HV, or total growth over the year, does not differ according to latitude, consistent with an equal total annual daylight hour exposure. Growth appears to speed up during times of greatest daylight exposure and slow down during periods of darkness, but overall has a consistent yearly “program,” possibly at the level of the growth plate. In a recent summary paper regarding evolution of the eye, Lamb describes the primitive eye, from which the current mammalian eye evolved 600 million years ago, as a light-sensitive organ whose function was that of maintaining circadian and seasonal rhythms [8]. The modern retina communicates via neural tracts with the pineal gland and regulates melatonin secretion affecting circadian and seasonal rhythms [9,10]. Pinealectomy eliminates daylight-entrained circadian patterns [11]. Melatonin has been implicated in gonadotropin regulation and more recently glucose metabolism/insulin sensitivity [12-15]. Recently, GH response to GH-releasing hormone and spontaneous GH secretion in sheep were demonstrated to be greater during a long versus short photoperiod, and GH amplitude was reduced by melatonin administration [16]. Effects of daylight hours on energy metabolism, other signaling at the level of the growth plate, or even GH secretion that were not identified in previous studies due to small numbers of patients evaluated may play a role. In plants, DNA methylation is involved in the photoregulation of flowering [17]. Melatonin may also induce epigenetic changes in DNA expression [18]. The observation that seasonal variation persists with continuous exogenous GH therapy, suggests that GH, if involved, is one of several factors.

In our analysis, BMI SDS at the start of each interval was associated with the HV of that interval. It is possible that weight gain during less active winter months in northern climates facilitates increased growth rate in summer. In the southern US, children’s activity level and weight gain are more similar year-round, and could account for less seasonal variability. This hypothesis was not supported by the finding that BMI SDS at the start of the HV interval was unrelated to the number of daylight hours (*P* = 0.28).

Our study was limited by the number of patients in the NCGS database who were measured at every 3 monthly intervals. Patients measured at 6 monthly intervals or longer were not included. Also, we do not have IGF-1 levels to correlate with 3 monthly HV intervals. 25-hydroxyvitamin D levels, a reflection of nutrition and ultraviolet light exposure, were also not assessed.

## Conclusion

In summary, there is seasonal variation in growth rate in children with IGHD during rhGH therapy that is associated with number of daylight hours and is greatest in the first year of therapy. The effect is modest and, except in extreme latitudes, is unlikely to interfere with short-term assessment of efficacy of a trial of rhGH. This observation confirms that rate of growth in children is not constant during the calendar year and underscores the need to measure HV over a 12-month period for an accurate assessment.

## Abbreviations

BMI: Body mass index; GH: Growth hormone; HV: Height velocity; IGF-1: Insulin-like growth factor 1; IGHD: Isolated growth hormone deficiency; NCGS: National Cooperative Growth Study; rhGH: Recombinant human growth hormone; SD: Standard deviation; SDS: Standard deviation score; US: United States.

## Competing interests

Dorothy Shulman has no competing interests to disclose. JF is a paid consultant to Genentech and to Ipsen. JF does not directly own stocks in any such organization although he owns mutual funds that have ownership in such stocks. JF does not hold nor is he currently applying for any such patents. JF has no other financial competing interests. BL is a paid consultant to Genentech. BL does not hold nor is she currently applying for any such patents. BL has no other financial competing interests.

## Authors’ contributions

DS conceived the study design and outline of the data to be analyzed; wrote the initial and final drafts of the paper; and organized the references. JF was the statistician that performed the analyses for the manuscript after consultation with the other authors; contributed to the text of the manuscript and reviewed all of the contributions to the manuscript from the other authors.; and read and approved the final manuscript. BL was responsible for determining the focus and type of data to be extracted and analysed from the NCGS database; contributed to the design and content presentation of the data; reviewed the contribution of the statistician and organized the data presentation; and contributed to the review of the final manuscript. All authors read and approved the final manuscript.
